# An Integrative View of the Role of *Lachancea thermotolerans* in Wine Technology

**DOI:** 10.3390/foods10112878

**Published:** 2021-11-21

**Authors:** Javier Vicente, Eva Navascués, Fernando Calderón, Antonio Santos, Domingo Marquina, Santiago Benito

**Affiliations:** 1 Unit of Microbiology, Genetics, Physiology and Microbiology Department, Biology Faculty, Complutense University of Madrid, Ciudad Universitaria, S/N, 28040 Madrid, Spain; javievic@ucm.es (J.V.); ansantos@ucm.es (A.S.); dommarq@bio.ucm.es (D.M.); 2 Department of Chemistry and Food Technology, Polytechnic University of Madrid, Ciudad Universitaria, S/N, 28040 Madrid, Spain; eva.navascues@upm.es (E.N.); fernando.calderon@upm.es (F.C.); 3 Pago de Carraovejas, Camino de Carraovejas, S/N, 47300 Valladolid, Spain

**Keywords:** *Lachancea thermotolerans*, lactic acid, wine, acidity, volatile, evolution, transcriptomic, metabolism regulation, lactate dehydrogenase enzymes

## Abstract

The interest in *Lachancea thermotolerans*, a yeast species with unusual characteristics, has notably increased in all ecological, evolutionary, and industrial aspects. One of the key characteristics of *L. thermotolerans* is the production of high quantities of lactic acid compared to other yeast species. Its evolution has mainly been driven by the influence of the environment and domestication, allowing several metabolic traits to arise. The molecular regulation of the fermentative process in *L. thermotolerans* shows interesting routes that play a complementary or protective role against fermentative stresses. One route that is activated under this condition is involved in the production of lactic acid, presenting a complete system for its production, showing the involvement of several enzymes and transporters. In winemaking, the use of *L. thermotolerans* is nowadays mostly focused in early–medium-maturity grape varieties, in which over-ripening can produce wines lacking acidity and with high concentrations of ethanol. Recent studies have reported new positive influences on quality apart from lactic acid acidification, such as improvements in color, glutathione production, aroma, malic acid, polysaccharides, or specific enzymatic activities that constitute interesting new criteria for selecting better strains. This positive influence on winemaking has increased the availability of commercial strains during recent years, allowing comparisons among some of those products. Initially, the management of *L. thermotolerans* was thought to be combined with *Saccaharomyces cerevisiae* to properly end alcoholic fermentation, but new studies are innovating and reporting combinations with other key enological microorganisms such as *Schizosaccharomyces pombe*, *Oenocous oeni*, *Lactiplantibacillus plantarum*, or other non-*Saccharomyces*.

## 1. Introduction

The interest in non-*Saccharomyces* applications in the wine industry during recent years has increased exponentially, and the number of scientific publications regarding the main species has increased accordingly [1]. Even so, in-depth study of all of the biological aspects surrounding this species has not followed or accompanied this interest. These studies, concerning the metabolic characterization of the species, as well as other evolutional and ecological aspects, may allow a better comprehension of the species and could serve to allow the rational selection of strains and to design new fermentative strategies.

The most studied non-*Saccharomyces* species are *Torulaspora delbrueckii*, *Lachancea thermotolerans*, *Schizosaccharomyces pombe*, *Metschnikowia pulcherrima*, *Pichia kluyveri* and *Hanseniaspora uvarum*. *L. thermotolerans* is the most reliable biological option for increasing the acidity and reducing the pH of wines from warm viticultural areas [2,3,4,5]. This is because of its unique ability to generate lactic acid from sugar metabolism during alcoholic fermentation [6]. This ability makes *L. thermotolerans* deserving of a section in all reviews dealing with non-*Saccharomyces* topics in winemaking. Since 2018, when the first review regarding *L. thermotolerans* in the wine industry was written [2], several reports have been published [7,8,9]. Until now, most reviews have focused on the application of *L. thermotolerans* in wine fermentation, showing some conclusions, establishing possible selection criteria, and proposing further challenges. Other reviews have focused on the beer industry [10]. Nevertheless, the reviews have not related these phenotypic characteristics to their biological basis.

Researchers have made important advances in the elemental biology of *L. thermotolerans.* The first studies analyzing the diversity among species [11] and the latest study on, not only the intraspecific diversity, but the phylogenetic relationship between them [6], have highlighted low intraspecific diversity. *L. thermotolerans* inhabits different environments. Among them, enological environments have been highlighted as the best source of isolation [9] due to the large number of strains that have been isolated there. Other environments, such as juices, that present high sugar concentrations are also efficient isolation niches. This agrees with the essential characteristics of *L. thermotolerans* since the species shows an extreme tolerance to high osmotic pressures and can grow in sugar concentrations up to 60% (*p*/*p*) [12]. The influence of these niches of isolation may have a great impact on the evolution of the species [6], influencing intraspecific diversity. Those strains from enological environments seem to be more adapted to those conditions than those from other niches, which directly influences the enological performance of a certain strain [6].

An increase in the number of accessible commercial wine strains has been accompanied by an increase in the scientific interest in *L. thermotolerans*. In 2018, there was only one available commercial strain of *L. thermotolerans* [2] sold alone or combined with other non-*Saccharomyces* strains. Today, there are seven strains from different manufacturers [13]. Two recent studies compare the three most popular available commercial strains [14,15], with an additional study comparing only two of them [16]. However, on some occasions, the results are contradictory, and the performance could depend on additional factors that are different from yeast strain parameters.

In 2018, it was possible to study combinations between *L. thermotolerans*, *S. cerevisiae* [2] and *S. pombe* [17]. Since then, most studies have combined *L. thermotolerans* with *S. cerevisiae* in order to ensure proper alcoholic fermentation. These studies allow us to review previous conclusions, test new strains with better performance, and study the influence of the new parameters. A new trend in winemaking is to combine *L. thermotolerans* with other microorganisms with enological interest. Some articles have studied new parameters, such as mannoproteins and polysaccharides production, in combinations of *L. thermotolerans* and *Schizosaccharomyces pombe* [18], achieving the desired microbial stabilization from a malic acid point of view directly after alcoholic fermentation while acidifying wine [19]. Recent works have studied combinations with *O. oeni* [15,20] in order to achieve microbial stability and to avoid difficult malolactic fermentations under difficult scenarios while acidifying wine. Other studies have investigated multistarters that include *L. thermotolerans* and other non-*Saccharomyces* strains such as *Torulaspora delbrueckii* in order to improve flavor and volatile compound composition [21]. A new combination is a mixed fermentation between *L. thermotolerans* and *Lactiplantibacillus plantarum* (formerly *Lactibacillus plantarum*) [20]. This new biotechnology avoids possible collateral effects produced by the heterofermentative metabolism of *O. oeni* over sugar during long alcoholic fermentations.

Recent studies have explored the influence of *L. thermotolerans* on parameters different from lactic acid acidification but that are still of great interest in modern enology, such as aeration influence [22], glutathione production [23], ethanol reduction [19], sulfur dioxide reduction, [14], polysaccharides production [18], and nitrogen demand.

The aim of the present review is to update the current scientific bibliography regarding *L. thermotolerans*, review the ecological, evolutional, and molecular information regarding this species, compare the commercial offerings, study new fermentation combinations, and synthesize the most important selection parameters.

## 2. Genomics, Ecology, and Evolution

The incongruity of the results regarding the morphology of vegetative cells, sexual states, and the physiological tests to assess a yeast isolate of a certain genus or family has forced the development of other approaches in yeast systematics. In early studies, single-gene sequences were used for a taxonomical assignation, but later, analysis of a combination of multiple-gene sequences was employed. These novel approaches allowed a more accurate methodology to establish the phylogenetic relationships among yeast. Using these approaches, Kurtzman and Robnett [24], analyzing different molecular targets such as 18S rDNA, ITS1-5.8S rDNA-ITS2, 26S rDNA, translation elongation factor 1 α, actin-1, RNA polymerase II nuclear genes, and cytochrome oxidase II (mitochondrial gene), revealed the presence of different clades among the *Saccharomyces* complex. One of these clades comprised several yeast species from different genera, separate from the clade that they were supposed to belong to (Clade 10: *Zygosaccharomyces cidri*, *Z. fermentati*, *Kluyveromyces thermotolerans*, and *K. waltii*). In addition, all of these species were protoploid *Saccharomycetaceae*, which reveals the divergence of this clade before the whole-genome duplication (WGD) occurred 100–150 million years ago [25]. Further studies have described a new genus that englobes a list of different species, named *Lachancea*, in honor of Dr. Marc-André Lachance from Western Ontario University, London, ON, Canada [12]. Nowadays, the genus englobes around 11 different species, *L. thermotolerans* being the type species.

The first genome sequence of the species *Lachancea thermotolerans* was in 2009 by the Genolevures Consortium [26]. This first genome showed large-scale compositional homogeneity, the GC content variations being linked or limited to local differences between intergenic regions (lower GC content), protein-coding regions (higher GC content), and genes for noncoding RNAs (the highest GC content) [27]. Since then, only two strains have been sequenced for differential lactic acid production [28].

Mitochondrial DNA has been used widely to study genomic evolution within and between different yeast species. Before the *Lachancea* genus settlement, the study of the restriction patterns of the mitochondrial DNA of several strains of *Kluyveromyces thermotolerans* showed a high homology among the species, despite the geographic and niche origin of the strains. Originally, this was related to the recent origin of the species, to the action of any evolutionary force, or a low mtDNA substitution rate [11].

Analysis of the mitochondrial genome of different species of the *Lachancea* genus has revealed syntenic genomes exhibiting a similar architecture. The protein-coding sequences indicate a high level of similarity, in contrast to the significant variation in the size and composition shown by intergenic regions [25]. Already working with *Lachancea thermotolerans*, this observation was later confirmed to be maintained intraspecifically as well. Freel and co-workers sequenced the mitochondrial genomes of 50 strains from different ecological niches and geographic origins [29]. Mitochondrial genomes have extremely low intraspecific divergence rates (π = 0.0014), being low in the intergenic sequences and extremely low in the coding regions. Additionally, these genomes have undergone few rearrangements during the evolution of the species. Regarding this information, the different studies have confirmed the hypothesis that this species, as well as the genus, has gone through a strong purifying selection or has an exceptionally low mutation rate as far as the mitochondrial genome is concerned.

Nevertheless, analysis of intraspecific diversity is essential for deepening the understanding of the divergence process that has occurred in a species. Because of the low differentiation of the mitochondrial DNA and the fact that nucleolar genes evolve in different ways and at different rates, these have been the target for determining the evolution process that *L. thermotolerans* has undergone.

The interest in intraspecific diversity, which has a direct influence on the ecological role that a yeast species play in a particular environment, has increased in recent years. Since *L. thermotolerans* is a non-*Saccharomyces* species with a great impact on and interest in the wine industry, several studies regarding the effects of this intraspecific variation have been carried out.

The first study was carried out by Banilas and co-workers and described the influence of the location in the population structure in strains from different vineyards [30]. A study of several microsatellites dispersed along the nuclear genome of the species in several *L. thermotolerans* strains from two different locations in southern Greece revealed a strong geographical clustering. In each zone, unique molecular patterns were found to be locally conserved; additionally, in each location, the profiles of the strains associated with a certain vineyard were clustered together.

This first study revealed the influence of the location on the species structure; nevertheless, further studies confirmed the influence not only of the location but also of the ecological niche of the isolation [31]. A study of a great number of strains of *L. thermotolerans* from different niches (natural and anthropic) dispersed along all of the glove revealed several groups clustering according to their ecological origin. Among the identified “natural” or “wild” clusters, the origin of the sample mainly influenced the distribution. What is more, samples with the same origin but collected 20 years apart presented similar genotypes. This result could confirm the domination of a certain genotype in a specific niche, which supports the idea of niche appropriation due to specific metabolic traits and the evolutionary differentiation to specific environmental conditions, as some domesticated strains have revealed [6].

Isolates from anthropic origins show signs of selection, clustering into two separate groups with common ancestors. This reveals the influence of at least two different domestication processes. These strains are not niche-associated, which means that all of the strains come from a single anthropic niche cluster regardless of the source of isolation (wine, milk, agave, fruit, etc.) and geographical origin (e.g., strains from Europe and America cluster together). Sometimes, when there is no limitation between environments, differentiation is not plausible, and the same cluster contains strains from natural and domesticated environments [31].

*L. thermotolerans* is, in fact, one of the most fermentative yeasts among the non-*Saccharomyces* group. This feature, a winning trait due to the ability to convert sugars into ethanol under both aerobic and anaerobic conditions, allows it to conquer different niches because of the increased metabolic capacity. *L. thermotolerans* is among the species that present the Crabtree effect, so under high glucose conditions, this yeast presents aerobic alcoholic fermentation [32]. The metabolic flux under these conditions in this species is still partially unknown, and to clarify this point, carbon metabolism and expression studies are necessary.

Different issues have been studied in order to decipher the role of *L. thermotolerans* under fermentative conditions, among which the influence of different genetic clusters [6], the transcriptomic/proteomic profiles [33,34,35], and the molecular basis of such an uncommon activity as lactic acid production [28,36] have been investigated.

Despite *L. thermotolerans* presenting some signs of domestication and allopatric differentiation, the capacity of proliferation under winemaking conditions may reveal the absence of an important niche specialization process [6]. Different isolates present the same clustering when studying the fermentative profile as previously obtained by microsatellites [31]. Despite this differentiation, the general characteristics seem to be common in the species, regardless of origin, including the low fructophilic character, high fermentation ability, and the production of lactic acid without an increase in acetic acid. Other features indicate the distinct phenotypic performance and are extremely variable among strains, the production of volatile compounds being the most remarkable one.

## 3. Molecular Regulation of Fermentation

**Figure 1:**
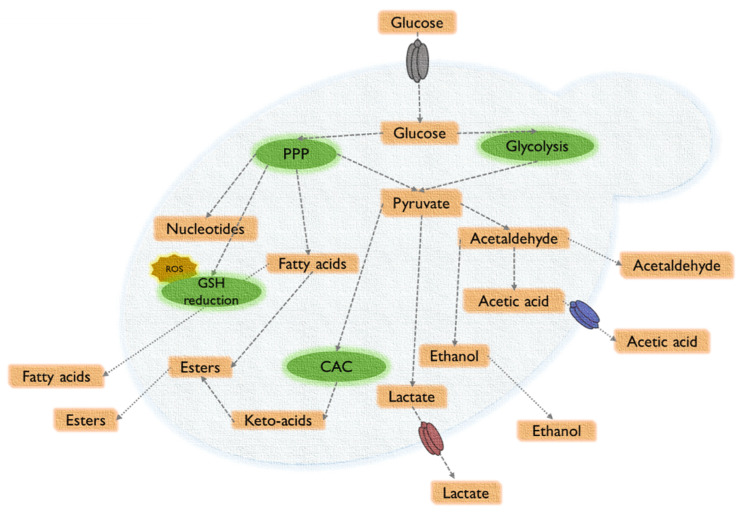
Main metabolic routes in *L. thermotolerans* under fermentative conditions. Metabolic processes are indicated in green, while intermediate or final metabolites are indicated in yellow. The presence of specific transporters is indicated in the scheme. PPP: Pentose Phosphate Pathway. GSH: Glutathione. ROS: Reactive Oxygen Species (Oxidative stress) CAC: Citric Acid Cycle. Source: self-made.

Despite the behavior of the species under different conditions and the intraspecific diversity, the analysis of the mechanisms that allow the development of yeast under real fermentative conditions is essential to properly know the influence of this species both in the fermentative process and on other species. Figure 1 summarizes the main metabolic routes under fermentative conditions.

Alcoholic fermentation, which transforms grape must into wine, is not a single-species process. In most cases, a strong fermentative yeast such as *S. cerevisiae* is essential to complete the process. The molecular basis of the influence of *L. thermotolerans* on *S. cerevisiae* is essential for understanding how fermentation progresses. The proteomic profile of *S. cerevisiae* under co-fermentation with *L. thermotolerans* shows different signs of stress under the presence of *L. thermotolerans*, displayed with several fighting and defensive mechanisms to keep itself dominant in the first stages of fermentation. As fermentation progresses and the *L. thermotolerans* population decreases, *S. cerevisiae* increases its enzymatic activity to allow better survival [33]. In the first stages, *S. cerevisiae* increases the nutrient availability and uptake by synthesizing specific proteins that allow the consumption of secondary carbon and nitrogen sources (e.g., aminomethyltransferases for glycine exploitation), as well as others for stress resistance (e.g., heat shock proteins and methionine), and apoptosis repression. On the contrary, in advanced stages of fermentation, *L. thermotolerans* cells induce protein synthesis (mainly those involved in translation, ribosome biogenesis, and aminoacyl-tRNA synthetases) and repress the stress response [33].

Wine fermentation is an oxygen-limited process in which yeasts may suffer from hypoxic conditions and ethanol or osmotic stresses. *L. thermotolerans* seems to be more affected by low oxygen availability than by the other conditions. Shekhawat and co-workers analyzed the RNA-seq profile of yeast under oxygen deprivation in order to explain the mechanisms by which the yeast faces this stress, both in pure and mixed fermentations [35].

In single cultures under anaerobic conditions, the main upregulated genes in *L. thermotolerans* are those involved in glycolysis and fermentation; on the contrary, those genes involved in the pentose phosphate pathway and the citric acid cycle, as well as the main biosynthetic routes (i.e., amino acids and nucleosides synthesis), are downregulated [35]. Among the genes involved in glycolysis and fermentation are those that codify osmotic sensors, transporters, and the main involved enzymes. Nevertheless, a group of three genes, coding for different lactate dehydrogenases (LDHs), are among the most upregulated and allow one of the most valuable technological applications of *L. thermotolerans* in wine fermentation—the production of lactic acid. The high expression (as well as its codification) has been linked to a lack in the expression of alcohol dehydrogenase enzymes (only two are expressed in comparison to other yeasts that express up to seven) and to be an alternative pathway to obtain reductive power and to maintain the redox balance. The fermentative process, in combination with oxygen depletion, influences other processes. The main influenced processes are those involved in restoring the damage caused by ethanol: lipid metabolism, cell wall modification, and intracellular homeostasis (ions, oxidative stress, etc.), as well as those involved in autophagy when cells are extremely damaged [35].

In mixed fermentations, anaerobic conditions force *L. thermotolerans* to exhibit a stronger modification in its expression profile than that displayed by *S. cerevisiae* [34]. This change in culture conditions has a significant influence on the carbohydrate metabolism and lipid biosynthesis in *L. thermotolerans*. On the contrary, under anaerobic conditions, when comparing axenic to mixed cultures, the most enriched processes are those related to nutrient uptake, mainly filamentous growth (as a response to starvation) and iron homeostasis. Nevertheless, under these conditions, the most influenced cellular component is the cellular wall upon activating genes for biogenesis and stabilization by β-glucan synthesis. Taken together, the interaction between mixing and anoxia triggers a strong response in *L. thermotolerans*, showing increased signals of cell aggregation, cell death, and osmotic and oxidative stresses. The stress to which cells are exposed alters the main carbon metabolism in *L. thermotolerans* by its redirection to PPP from glycolysis as a possible tool for oxidative stress protection [37]. All genes related to the phenylalanine metabolism and phenylethanol are upregulated, which is in agreement with previous studies regarding the compositional analysis of must, showing that under mixing conditions, the production of phenylethanol is increased compared to single fermentations using *L. thermotolerans* [2,19].

The fermentative conditions force several mechanisms in the sugar metabolism, the most remarkable of which is the increased activity of LDH enzymes, as described before. Despite the production of lactic acid being a general characteristic of the species, the amount produced is extremely variable. The lactic acid metabolism in *L. thermotolerans*, as well as its real (ecological and evolutionary) relevance, is far from being understood because of the lack of molecular information and prospective studies based in a major universe. Nevertheless, different conditions that may cause a differential production of lactic acid among strains have been studied, including the number and sequence of coding genes for LDH, transcriptional regulation of the enzyme synthesis, and extracellular transport of lactate.

In silico analysis has presented three different LDH coding genes in *L. thermotolerans*. One of them, designated as *ldh1*, is located in chromosome D, while the two others, *ldh2* and *ldh3*, are in tandem in chromosome G. Expression analysis of these three genes under fermentative conditions by RT-qPCR has shown that, apparently, only one of them (*ldh2*) is responsible for the different lactic acid production between strains, with *ldh1* and *ldh3* showing similar expression profiles in all strains independently of their lactic acid production under fermentative conditions. Interestingly, this increased lactic acid activity is not accompanied by a decreased expression of alcohol dehydrogenase (ADH) genes [36]. A deeper study of the coding regions of the enzymes, including their promoter sequences, showed important differences between high and low lactic acid-producing strains [28]. Gatto and co-workers showed that despite the differences in the aminoacidic sequence between the three enzymes, the key sites in the proteins might not be affected. Phylogenetic analysis of the promoters showed some single-nucleotide mutations (in the TATA box) among strains, which can seriously affect the promoter activity. Among the enzymes, ldh1 and ldh2 presented more similar upstream regions despite the studied strain, whereas ldh2 and ldh3 presented a similar promoter core sequence linked to increased activity. The study of transcription binding factors, which are usually linked to environmental stress responses, in the three enzymes showed a specific profile for ldh2 linked to increased activity. The expression of ldh1 and ldh2 during the fermentation process presented a similar profile, being more active in the early stages of fermentation, and presenting decreased activity at the end. In high-producing strains, the activity of both enzymes was higher; nevertheless, the differences in the expression of ldh2 were extremely marked. Interestingly, at the end of the fermentation process, both enzymes showed a higher expression level in the low-producing strains.

Changes in the regulatory machinery may allow a better transcriptional rate of the LDH coding genes that, combined with the differential expression, is linked to major activity. Despite this, it is still unknown if the expression differences in these genes are associated with functional ones.

The third element that may play an important role in lactic acid metabolism may be the presence of transporters in charge of its extrusion to the extracellular environment. If a huge amount of lactic acid is accumulated inside cells, it can cause feedback inhibition of the LDH enzyme; thus, transporters for monocarboxylic acids across the membrane are essential for lactic acid production [38,39,40]. In the case of *L. thermotolerans*, no lactic acid transporter has been confirmed; nevertheless, there are some candidates based on its homology to lactate permeases encoding genes in *S. cerevisiae*, *jen1,* and *ady2* [28]. Both genes are present in all studied strains, *jen1* being a single copy and *ady2* twice encoded in all strains despite their lactic acid production. It remains unknown if the presence of two copies of the same gene is linked to increased activity since the type of strain, *L. thermotolerans* CBS 6340, which is a low lactic acid producer, presents only one copy of each of these homologs. In engineered *S. cerevisiae* strains, the increased number of copies or expression levels is not related to a higher lactic acid production [40]. Gatto and co-workers also analyzed the aminoacidic sequence of both transporters, and in the case of *jen1*, it is more similar in those strains that exhibit a lower production of lactic acid to those that present a higher one. The presence of these two genes confirms the presence of a complete lactate production system.

## 4. Impact of *L. thermotolerans* on Different Wine Quality Parameters

### 4.1. Ethanol

Modern winemaking is looking for new technologies that are able to reduce the final ethanol concentration of wines from warm viticulture areas. The first studies regarding *L. thermotolerans* focused on the ability to ferment as much as possible so as not to have a big dependency on the most fermentative yeast species, such as *S. cerevisiae* [19]. The fermentative power of *L. thermotolerans* varies from 4.24% to 10.6% (*v*/*v*) depending on the strain [2,6,41] (Table 1). New studies have reported that, depending on the strain variability and physicochemical conditions, *L. thermotolerans* can ferment up to 13.6% (*v*/*v*) (Table 1), with an ethanol yield from 0.34 to 0.4 g/g and generating lactic acid concentrations that vary from 1.8 to 12 g/L with an average value of 5.8 g/L [6]. These results suggest that with proper management, in the near future, specific *L. thermotolerans* strains could complete alcoholic fermentation in regular wines by themselves without help from other, more fermentative yeasts.

An interesting approach of *L. thermotolerans* includes the ability to generate wines with a lower final ethanol concentration in warm viticulture areas for combined fermentations compared to *S. cerevisiae* pure fermentations [19]. Several authors have recommended using *L. thermotolerans* in grape juices that suffer from a lack of acidity and a high potential final ethanol because of high sugar concentrations. *L. thermotolerans* is less efficient than *S. cerevisiae*, decreasing the ethanol concentration in a range from 0.1% to 1.6% (*v*/*v*) [36,44]. Higher decreases, down to 3% (*v*/*v*) less, take place in combined fermentations with *S. pombe* [45].

The fact that *L. thermotolerans* presents a weaker Crabtree effect than *S. cerevisiae* primarily explains ethanol reduction, which favors the respiratory metabolism. Under fermentative conditions, the other important factors in *L. thermotolerans* are the production of lactic acid, yeast biomass, glycerol, and pyruvic acid.

### 4.2. Glycerol

Previous studies have typically reported mixed fermentations between *L. thermotolerans* and *S. cerevisiae* to be higher in glycerol than the *S. cerevisiae* control [2]. Such differences are moderate, varying from 0.29 to 0.93 g/L. The difference between strains is approximately 50% for 94 strains [6] and 20% for 13 strains [41], which matches the glycerol yield that varies from 0.02 to 0.047 g/g [6].

### 4.3. Organic Acids and pH

The lactic acid production is highly variable depending on the conditions and strain used. Previous works have reported maximum levels in lactic acid production between 9.5 and 12 g/L, which has great importance in pH, reducing it down to 0.59 units [2,15] [6]. Despite these data, some studies have reported strains that produce only approximately 0.69 g/L [2,41,46]. A previous study reported a lactate yield varying from 0.0086 to 0.0658 g/g. The achieved acidifications and pH reductions related to the use of *L. thermotolerans* are a serious alternative to other acidification technologies such as potassium ion reduction, synthesized acid addition, or early harvest.

The consumption of malic acid by *L. thermotolerans* may reduce its concentration from 10% to 50% [2,14,47]. This feature may affect malolactic fermentation, making it shorter, which winemakers appreciate because of the reduction in time and risks. Contrarily, only one study has reported an *L. thermotolerans* strain (LL12_056) able to increase the malic acid concentration from an initial grape juice of the Chardonnay grape variety, from 3.8 to 4.1 g/L of malic acid, although other studied strains have been shown to consume original malic acid down to 3 g/L [6]. These results suggest that a few *L. thermotolerans* strains could possess the ability to generate small amounts of malic acid similar to *S. cerevisiae* species [48]. This ability could be of interest, especially in white wine production, as most commercial white wines do not need malic acid stabilization and could increase the acidification effect of *L. thermotolerans* lactic acid.

*L. thermotolerans* may produce succinic acid in high concentrations, with an average value among 13 strains of 0.5 g/L, compared to no succinic acid production in the *S. cerevisiae* control [41]. Contrarily, another study reported succinic acid varies from 13 to 78 mg/L [6].

### 4.4. Acetic Acid

Previous studies have reported that *L. thermotolerans* produces low concentrations of acetic acid, below 0.24 g/L, but with a high strain variability of up to 50% with variations from 0.03 to 0.58 g/L [2,6,41]. The acetate yield may vary from 0.3 to 1.53 mg/g [6].

### 4.5. Acetaldehyde

Previous studies have reported *L. thermotolerans* as a lower producer of acetaldehyde than *S. cerevisiae*, producing final acetaldehyde concentrations that vary from 15 to 19 mg/L in pure fermentations [2]. Sequential fermentations usually also show lower final concentrations than pure *S. cerevisiae* controls. However, one scientific article reported the opposite effect for sequential fermentations [2]. A new study that performed five sequential fermentations between different *L. thermotolerans* strains and one *S. cerevisiae* strain reported that all sequential fermentations showed significant differences compared to the *S. cerevisiae* control [14], as well as in other cases [49]. In other cases, two strains of *L. thermotolerans* reduced the acetaldehyde concentration by 17 and 12 mg/L compared to the *S. cerevisiae* control. However, one strain did not show significant differences [42].

### 4.6. Anthocyanins, Polyphenols, and Color

The increase in red colorations of anthocyanins takes place when the pH decreases because lactic acid formation improves the color intensity [19]. The increases in color intensity and total anthocyanins related to the influence of *L. thermotolerans* vary from 8% to 10% [2]. However, sometimes, the color intensity diminishes, probably because of the high strain variability in anthocyanin absorption for this parameter, similar to what occurs for *S. cerevisiae* [19]. A new study performed in the Sangiovese red grape variety showed that a sequential fermentation between *L. thermotolerans* and *S. cerevisiae* has a 14% higher concentration in final total anthocyanins and 9% more in total polyphenols than the *S. cerevisiae* control [49]. The same study observed significant differences in color perception, where sequential fermentation of *L. thermotolerans* scored 1.5, and the *S. cerevisiae* control scored 1.25, which represents an increase of 16%. Other studies have reported a lower increase of around 12% when comparing a sequential fermentation involving *L. thermotolerans* and the *S. cerevisiae* control [20].

### 4.7. Glutathione

Glutathione possesses anti-oxidative properties that are interesting in modern winemaking for avoiding detrimental changes in color and oxidative aromas. It also allows a reduction in the addition of sulfur dioxide, which is a hazardous compound, especially for specific groups at risk, such as people who suffer from asthma, and its legal limits have decreased in recent years [50]. Some non-*Saccharomyces* strains have been recently reported to be powerful producers of glutathione [23]. Three strains of *L. thermotolerans* contain glutathione in concentrations varying from 0.205 to 0.537 nmol/mg cell, while the *S. cerevisiae* control shows 0.095 nmol/mg cell. *L. thermotolerans* produces 2–5 times more glutathione than *S. cerevisiae*. Nevertheless, the strain variability in glutathione production is 60%. For this reason, the production of glutathione may be an interesting selection parameter for future selection processes regarding *L. thermotolerans* strains.

### 4.8. Polysaccharides

*L. thermotolerans* has been reported to be a higher producer of polysaccharides than *S. cerevisiae* by up to 23%, but with a high strain variability of up to 60% [2,49]. Recent studies focused on this parameter have reported *S. cerevisiae* wines without differences between the sequential fermentation and the *S. cerevisiae* control [18]. It is interesting to mention that among the sequential fermentations of seven different non-*Saccharomyces* and *S. cerevisiae*, *L. thermotolerans* was the highest producer of total polysaccharides. These results show the importance of introducing this parameter in selection processes.

### 4.9. Enzymatic Activity

The enzymatic profile of *L. thermotolerans* is extremely variable [51]. All assayed strains have been shown to be positive for esterase-lipase and leucine-A, while esterase, lipase, valine, cysteine-A, and β-glucosidase are strain-dependent. Other activities, such as protease, polygalacturonase, glucanase, xylanase, and cellulase, are rare in *L. thermotolerans* [51,52]. This high strain variability in specific enzymatic activities is of great interest in the *L. thermotolerans* strain selection processes as well.

### 4.10. Aroma Composition

*L. thermotolerans* usually produces wines with lower amounts of total higher alcohols; however, the opposite can take place due to a high strain variability of approximately 40% [2,14,44] and the aeration conditions of the fermentation [22]. All previous works have reported fermentations involving *L. thermotolerans* to produce higher concentrations of total esters (ethyl and acetate esters) than *S. cerevisiae* controls, mainly because of ethyl lactate [14,22,53]. Compounds such as fatty acid and terpenes are less studied. Under aeration conditions, the fatty acid content may increase [22]; otherwise, it seems to decrease [14]. As far as terpenes are concerned, *L. thermotolerans* does not influence [14,53]—or even increases [43]—the final concentration.

## 5. *L. thermotolerans* Commercial Strains Comparisons

Nowadays, the market offers seven different *L. thermotolerans* strains from three manufacturers [13,54]. Additionally, there is a commercial product named Melody^TM^ (Hansen, Horsholm, Denmark) that consists of a multistarter of three different yeast species, one of them being the *L. thermotolerans* Concerto strain.

Recent studies have compared some of the different available commercial strains (Levulia^TM^, AEB, Italy; Concerto^TM^, CHR Hansen, Denmark; Laktia^TM^, Lallemand, Canada) [14,15,16], showing interesting differences and even contradictory results regarding several enological parameters, depending on the study. According to all of them, sequential fermentation using *L. thermotolerans* and *S. cerevisiae* is more efficient than co-inoculation as far as lactic acid production is concerned. After studying the comparisons between commercial strains of *L. thermotolerans* fermenting in sequential fermentations, contradictory results have appeared. Such results could show that the commercial strains might perform differently depending on different factors, such as available nutrients, aeration conditions, or other unknown factors. Table 2 summarizes the observed parameters in the studies that have compared commercial *L. thermotolerans* strains.

### 5.1. Ethanol

Using commercial strains may slightly influence the ethanol concentration. Sequential fermentations between the Concerto or Laktia strains and *S. cerevisiae* achieves ethanol reductions of 0.9% (*v*/*v*) compared to the *S. cerevisiae* control, while fermentations involving the Levulia strain under these conditions do not show significant statistical differences [14]. All sequential fermentations consume all of the fermentative sugars. Another study reported that the commercial *L. thermotolerans* strains (Laktia, Levulia, and Concerto) slightly reduce the final ethanol concentration in sequential fermentations by 0.475, 0.33%, and 0.16% (*v*/*v*), respectively, compared to the *S. cerevisiae* control [15]. Pure fermentations using Concerto reach an ethanol concentration of up to 8% (*v*/*v*), while Laktia ferments up to 6.2% (*v*/*v*) [16].

### 5.2. Glycerol

The Concerto strain is the highest glycerol producer, up to 11.6 g/L, while Laktia and Levulia produce slightly lower final concentrations, with non-significant differences between them (both approximately 10 g/L) [14]. The *S. cerevisiae* control produces 30% less glycerol than the sequential fermentations between Concerto and *S. cerevisiae*. Other studies have reported non-significant differences for the three studied commercial *L. thermotolerans* strains in sequential fermentations, while the *S. cerevisiae* control produces 15% less glycerol [15].

### 5.3. Organic Acids and pH

The production of lactic acid, the most valuable virtue of *L. thermotolerans*, is extremely variable depending on the strain and fermentative conditions. The first study that compared three available commercial *L. thermotolerans* strains (Levulia, Concerto, and Laktia) fermented Merlot grape must via co-inoculation and sequential strategies. Laktia was the highest lactic acid producer, up to 5.8 g/L (pH drop of 0.35 units), while Concerto (pH drop of 0.15 units) and Levulia produced 41% and 82% less, respectively [14]. Another study performed on the Sauvignon blanc grape variety reported sequential fermentations of Levulia to be the highest lactic acid producer, generating up to 2.8 g/L and reducing the pH by approximately 0.23 units, followed by Laktia (46% less and pH drop of 0.1 units) and Concerto (83% less) [15]. In other studies, no differences in lactic acid production between Concerto and the *S. cerevisiae* control in pure fermentations using Airen must have been observed, while the use of Laktia allowed to reach up to 1.8 g/L of lactic acid [16].

Sequential fermentations involving Levulia and *S. cerevisiae* have not shown statistical differences to the *S. cerevisiae* control in total acidity, while sequential fermentations involving Concerto and Laktia showed increases of 37% and 44%, respectively [14]. On the contrary, other cases have not shown differences in the final total acidity concentrations in sequential fermentations involving Concerto and the *S. cerevisiae* control, while fermentations involving Laktia and Levulia showed higher final concentrations in 18% and 31%, respectively [15]. In pure Concerto fermentations, compared to the *S. cerevisiae* control, no difference took place, while the Laktia control increased the total acidity by 0.7 g/L in 1 L fermentations [16].

Similar malic acid degradations of approximately 50% took place for all of the studied commercial *L. thermotolerans* strains (Levulia, Concerto, and Laktia) in sequential fermentations with *S. cerevisiae*, while the *S. cerevisiae* control degrades 7% in Merlot wine with an initial malic acid concentration of 2.6 g/L [14]. These results are very promising for new strategies trying to generate stable wines from a malic acid point of view directly during alcoholic fermentation, avoiding difficult malolactic fermentations under specific scenarios [15,19,20]. However, another study performed in Sauvignon blanc must reported slight reductions of malic acid for the commercial strains that varied from 7% to 10%, with no differences with the *S. cerevisiae* control; in this case, the initial concentration of malic acid was significantly higher (4.72 g/L) [15].

No significant differences took place in the final concentration of succinic acid (around 3 g/L) among the commercial strains, although the *S. cerevisiae* control showed a lower final concentration of approximately 50%. Laktia showed the highest pyruvic acid production, up to 0.17 g/L, while Concerto and Levulia showed lower concentrations than *S. cerevisiae* (0.13 g/L) [14].

### 5.4. Acetic Acid

*L. thermotolerans* is generally a lower producer of acetic acid than the *Saccharomyces* genus [2,7]. However, *L. thermotolerans* possesses a strain variability of up to 50% in acetic acid production [2,46]. Slight differences have been reported in the final concentration of acetic acid for sequential fermentations involving the Laktia, Concerto, and Levulia strains. The acetic acid concentration varied from 0.10 to 0.13 g/L, while the *S. cerevisiae* control showed significantly higher concentrations that varied from 23% to 41% higher than the *L. thermotolerans* sequential fermentations [15]. Surprisingly, sequential fermentations involving the Levulia *L. thermotolerans* strain produced 0.29 g/L of acetic acid, while Concerto and Laktia fermentations produced 40% more acetic acid without significant differences between them; the *S. cerevisiae* control produced the lowest final concentration in acetic acid, approximately 50% less than the Levulia control [14].

### 5.5. Total Sulfur Dioxide

Levulia showed the lowest final concentration in total SO_2_ of 0.5 mg/L in sequential fermentations, while the *S. cerevisiae* control showed a concentration of up to 13.3 mg/L (96% higher) [14]. This effect could be related to the low production of acetaldehyde and pyruvic acid in the strain. Concerto and Laktia showed lower values than the *S. cerevisiae* control by 66% and 74%, respectively. Another study reported that Concerto, Levulia, and Laktia produce lower concentrations of total sulfur dioxide compared to the *S. cerevisiae* control by 65%, 61%, and 48%, respectively [15]. The *S. cerevisiae* control showed a final total sulfur dioxide concentration of 23 mg/L. Although in distinct order, the different studies agree that sequential fermentations involving commercial *L. thermotolerans* strains reduce the final concentration of SO_2_. This effect is of great interest for the production of wines with low concentrations of SO_2_ that are highly appreciated by some consumers.

### 5.6. Aroma Compounds

The commercial *L. thermotolerans* strains (Levulia, Concerto, and Laktia) produced higher concentrations in total esters and lower fatty acids than the *S. cerevisiae* control [14]. Although all *L. thermotolerans* strains produced higher total esters than the *S. cerevisiae* control, there were big differences between them, as Levulia produced 30% more esters than the *S. cerevisiae* control, while Concerto and Laktia produced 71% and 75% more, respectively [15]. Concerto was the highest producer in sequential fermentations of ethyl acetate, isoamyl acetate, and 2-phenylethyl acetate, while Laktia was the highest producer of ethyl lactate [14]. Another study also observed the commercial *L. thermotolerans* strains to produce higher concentrations of esters than the *S. cerevisiae* control, although with bigger differences between Laktia and Concerto [16]. The study reported Laktia to produce, in pure 1 L fermentations, 40% more esters than the *S. cerevisiae* control, while Concerto only produced 20% more. The study highlighted Concerto producing less ethyl acetate than Laktia by approximately 30%.

Levulia produced similar final higher alcohol concentrations to the *S. cerevisiae* control without significant differences, while Concerto produced 16% more higher alcohols and Laktia produced 14% less [14]. Another study reported Laktia and Concerto producing similar final concentrations of higher alcohols in pure fermentations of 1 L, while the *S. cerevisiae* control produced 31% less [16].

Laktia produced 77 mg/L of carbonylic compounds, while Concerto produced 39% less and the *S. cerevisiae* control 61% less [16]. The only study that reported results regarding fatty acids stated that all commercial *L. thermotolerans* strains produce lower concentrations than the *S. cerevisiae* control [14]. Concerto and Levulia showed similar concentrations of 39% and 43% lower than the *S. cerevisiae* control, while Laktia produced 65% less.

We can observe that the results are not homogenous regarding aroma compounds depending on the study, which clearly indicates that there could be other significant factors than this strain parameter.

## 6. *L. thermotolerans* Combinations with Other Microorganisms in Wine Technology

### 6.1. Saccharomyces cerevisiae

The first study that reported the use of *L. thermotolerans* in winemaking showed that although it is the best biological acidifier, it cannot completely ferment a wine of a regular degree of potential ethanol [55]. The wine reached a final ethanol concentration of 7.58% (*v*/*v*) and the *S. cerevisiae* control of 9.6% (*v*/*v*). Therefore, the next studies used strains of the *Saccharomyces* genus as a partner in order to ensure proper alcoholic fermentation [56]. Since then, most studies have employed mixed combinations between *L. thermotolerans* and *S. cerevisiae* in different modalities, such as mixed initial inoculations with different ratios or sequential fermentations, where *L. thermotolerans* ferments in the first few hours or days and after *Saccharomyces* ends its alcoholic fermentation [2]. The sequential modality is the one that allows *L. thermotolerans* to influence the final chemical composition of wine the most. This is because *Saccharomyces* usually inhibits the growth of *L. thermotolerans* as soon as it is inoculated. Table 3 summarizes the different results regarding lactic acid, pH, and total acidity of original works that used sequential fermentations between *L. thermotolerans* and *Saccharomyces*.

### 6.2. Schizosaccharomyces pombe

The combination of *L. thermotolerans* with another powerful fermentative yeast, such as *S. pombe*, allows avoiding performing malolactic fermentation in wines with high ethanol concentrations and high pH levels from warm viticulture areas or overripe grape varieties. In this combination, *L. thermotolerans* increases the acidity, generating lactic acid, while *S. pombe* consumes the unstable malic acid and ends the alcoholic fermentation. The result is a wine stabilized from a malic acid point of view and acidified during alcoholic fermentation. Therefore, the wine does not need to undergo malolactic fermentation after alcoholic fermentation.

The studies performed from 2015 to 2018 focused on the effect of this possible combination on basic enological parameters, aroma compounds, and color [19]. This strategy solves the specific problems of modern winemaking, such as a lack of acidity, biogenic amines, ethyl carbamate, or undesirable color losses that take place during classic malolactic fermentation. Color intensities may decrease during lactic acid bacteria malolactic fermentation. On the contrary, the combined use of *L. thermotolerans* and *S. pombe* allows maintaining color. The different studies justify the final difference in color because of a lower pH, a direct consequence of the lactic acid produced by *L. thermotolerans*, which increases the red color intensity and the formation of stable color forms such as vitisins by *S. pombe* [19].

### 6.3. Oenococus oeni

Some studies have introduced *Oenococus oeni* into the combinations between *L. thermotolerans* and the *Saccharomyces* genus in order to obtain stable wines from a microbiological point of view with a slight increase in the final lactic acid during alcoholic fermentation. This combination may increase the final lactic acid content by approximately 10% compared to the sequential *L. thermotolerans* control without lactic bacteria [20].

The regular use of this strategy usually comprises an initial co-inoculation between *L. thermotolerans* and *O. oeni* after 24 h, while *S. cerevisiae* is inoculated after 48–72 h. Recent studies have shown that some *L. thermotolerans* strains promote malolactic fermentation, while others inhibit it [15]. The presence of lactic acid itself inhibits malolactic fermentation. Levels over 6 g/L may completely inhibit malolactic fermentation [15]. These facts justify the recent interest in co-inoculations between *L. thermotolerans* and *O. oeni* at the beginning of alcoholic fermentation. However, this new fermentation modality may carry the risk of an increase of acetic acid when *O. oeni* metabolizes sugars [20]; this risk decreases if the alcoholic fermentation takes place properly, without stopping or becoming sluggish. Recent studies have reported increases in acetic acid compared to co-inoculation and classical sequential inoculation of *O. oeni*, varying from 0.03 to 0.2 g/L [15,20]. One study reported increases in acetic acid of approximately 50% for sequential classic malolactic fermentations after a sequential fermentation between *S. cerevisiae* and *L. thermotolerans* [43].

### 6.4. Lactiplantibacillus plantarum (Formerly Lactobacillus plantarum)

The newest trend introduces combinations of *L. thermotolerans* and other lactic bacteria different from *O. oeni* in order to avoid problems related to the heterofermentative metabolism of this species. Because of that metabolism, *O. oeni* is mostly inoculated once fermentative yeasts have metabolized all the sugar into ethanol. *Lactiplantibacillus plantarum* (formerly *Lactobacillus plantarum*) is an alternative lactic bacteria that possesses a homofermentative metabolism that allows it to be inoculated at the beginning of alcoholic fermentation without risk of sugar consumption. *L. plantarum* is an interesting option for combining with *L. thermotolerans* in grape juices that suffer from a lack of acidity and contain high initial concentrations of sugar that may originate from extended alcoholic fermentations longer than 21 days [20]. In such situations, *L. plantarum* metabolizes malic acid during the first stages of alcoholic fermentation, stabilizing the wine from a malic acid point of view. This method reduces the risk of undesirable spoilage microorganisms during difficult long alcoholic fermentation processes, as wine can be protected with sulfur dioxide, lysozyme, or chitosan once malic acid is eliminated. This combination always requires a final inoculation of a high fermentative yeast such as *S. cerevisiae* in order to end the alcoholic fermentation, especially in wines with a high potential ethanol concentration.

This approach reduces, by 18 days, the entire fermentation process and produces wines with improved quality parameters compared to the classical control fermented by classical sequential fermentation by inoculation of *S. cerevisiae* and *O. oeni* when alcoholic fermentation is finished [20]. The final concentration of lactic acid increases by 2.39 g/L with a pH reduction of 0.26 compared to the regular control. Other improvements are a slight increase in the glycerol concentration of 0.52 g/L. Decreases in ethanol and acetic acid have also been reported by approximately 0.37% (*v*/*v*) and 0.08 g/L, respectively. Regarding aroma compounds, this approach produces wines with higher concentrations of ethyl lactate due to the greater formation of lactic acid and the lower concentrations in diacetyl, as well as a significant increase of 37% in 2-phenyl ethyl acetate. Another study also reported a decrease in color intensity during the sequential malolactic fermentation by *O. oeni* that did not take place in the combined fermentation between *L. thermotolerans*, *L. plantarum*, and *S. bayanus*.

### 6.5. Other Non-Saccharomyces

*L. thermotolerans* may combine with other non-*Saccharomyces* strains in order to produce wines with different styles, and some researchers have considered the use of popular commercial *Saccharomyces* strains to increase the risk of producing standardized wines.

The commercial product Melody^TM^ (Hansen, Horsholm, Denmark) combines *L. thermotolerans* with *Torulaspora delbrueckii* and *S. cerevisiae* [54]. This product started to be commercialized in 2012 and was the first commercial starter that included several non-*Saccharomyces* yeasts. *L. thermotolerans* increases the acidity [54], *T. delbrueckii* increases tropical fruit aromas [66], and *S. cerevisiae* ensures the alcoholic fermentation is properly ended. One scientific study compared the combined multistarter Melody^TM^ [21] to a sequential fermentation of the Concerto^TM^ commercial *L. thermotolerans* strain with *S. cerevisiae* in Shiraz must. The multistarter showed a higher pH of 0.06 and 0.08 than the sequential *L. thermotolerans* control. The competence of *L. thermotolerans* combined with *T. delbrueckii* and *S. cerevisiae* explains these differences, as it results in a lower acidification effect than that when fermenting *L. thermotolerans* alone. The multistarter fermentation showed a lower final ethanol concentration of 0.2% (*v*/*v*). One trial reported no differences in acetic acid, while another reported a higher acetic acid production for an *L. thermotolerans* trial of 0.3 g/L. The production of acetate esters was reduced by around 21% in the multistarter fermentation; nevertheless, higher concentrations of 2-phenyl acetate and isoamyl acetate were observed. On the contrary, the concentration of ethyl esters increased by approximately 28%. No differences took place in terms of color intensity, phenolic substances, and non-bleachable pigment. The multistarter showed a lower concentration of anthocyanin by around 10% and a higher wine hue of 0.06.

Recently, an inoculum composed of *L. thermotolerans* and *T. delbrueckii* in a 30/70 ratio has been proposed [51]. A high implantation capacity characterizes the inoculum, improving wine quality parameters such as the glycerol and lactic acid concentrations significantly. Another proposal is ternary combinations of *L. thermotolerans* with *Metschnikowia pulcherrima* or *Hanseniaspora vineae* and *S. cerevisiae* [67]. A previous study reported an inhibitory effect of *H. vineae* on acidification, as well as a synergetic effect of *M. pulcherrima* on acidification and ethanol reduction.

Table 4 summarizes the enological impact, advantages, and disadvantages of the reported combinations of *L. thermotolerans* and other microorganisms in winemaking.

## 7. Grape Varieties, Warm Viticulture Areas, and Climate Change

The main focus of *L. thermotolerans* application is to improve the lack of acidity, which is one of the key problems affecting vineyards in warm viticulture areas. Other cooler regions could suffer similar problems in the future because of the evolution of climate change. Most studies focus on grape varieties that reach their maturity early. During the maturity process, vine plants synthesize sugar while combusting acids. The first acid to be degraded is malic acid, whose concentration is occasionally very low in very ripened or overripe grapes. In such cases, grapes can reach a pH of approximately four times the value considered high and may result in sensory and technical problems in winemaking. Mediterranean countries such as Greece [55,56], Italy [57,58], and Spain [60,61] reported the first applications of *L. thermotolerans*, which took place in the big locations of these warm viticultural countries. Climate change is generating problems in new regions that were not considered warm viticulture areas some years ago. The initial studies focused on the most planted national grape varieties from each country. In Italy, varieties such as Pinot Grigio [68] or Sangiovese [49,58] are the most studied alternatives, whereas, in Spain, Tempranillo for red grape plantations [17,60] and Airen [61] for white wine production are the most studied. Tempranillo possesses an early ripening effect (literal translation: “The earliest”), which reduces the potential risks related to long maturation. Airen is a very productive neutral variety whose wine possesses low levels of sugar and low acidity but with a great capacity for development in dry areas. The application of *L. thermotolerans* in Airen wines increases the acidity and esters, improving the acidity balance and fruit aroma, producing more complex wines than the classical neutral Airen [61]. Later studies were performed in Mencia, a red grape variety that presents late budding but early ripening. These properties, characterized by a short development cycle, make it ideal for producing red wine in Atlantic regions with short thermal integrals that were traditionally considered more appropriate to produce white wine. However, because of climate change, this variety has started to show overripening problems in some locations. *L. thermotolerans* showed interesting improvements when fermenting Mencia [47]. More recent studies have applied *L. thermotolerans* in early international red grape varieties, such as Merlot [14] and Syrah [69]. The other studied varieties are Riesling [53], Muscat of Alexandria [70], and Chardonnay [22] (Table 5). Table 5 clearly shows that most researchers have focused on *L. thermotolerans* applications in grape varieties of early–medium maturity in countries with warm viticulture areas that are more affected by climate change. This shows that other early- or medium-maturity varieties such as Pinot Noir, Malbec, and Tannat could also show positive effects in some regions that could suffer from climate change in the mid-future. Medium–late- or late-maturity varieties such as Carignena, Cabernet Sauvignon, Graciano, Bobal, Monastrel, and Petit Verdot remain to be tested, as overripening problems are less common. However, traditional varieties from cool Atlantic regions such as Treixadura have started to be tested with *L. thermotolerans* to mitigate the future effects of climate change [71].

## 8. Proposed Selection Parameters for *L. thermotolerans*

The latest studies that compared commercial strains to other selected strains showed that it is possible to select new strains that perform better than the commercially available ones in several parameters [14,15]. This shows how important it is to establish a proper selection method in order to improve the current commercial offerings and provide solutions to the modern challenges of winemaking.

Former reviews have established the three main *L. thermotolerans* strain selection parameters to be the production of lactic acid up to 9.5 g/L, acetic acid production down to 0.14 g/L, and fermentative power up to 10.4% (*v*/*v*) [2]. New studies have suggested updating the previous stabilized levels. According to recent studies, the lactic acid value can increase up to 11.5 g/L, and the acetic acid concentration can decrease below 0.1 g/L [15]. Other recent studies have suggested that with a proper strain selection and controlling the physiochemical conditions, it is possible to ferment up to 13.6% (*v*/*v*) [6,68].

New advances allow the introduction of new selection parameters. Interesting research performed regarding ethanol efficiency production [36] allows reducing the potential content of ethanol by 1% to 1.6%. This can establish a selection parameter for future studies. *L. thermotolerans* strains may be selected to reduce the malic acid content down to 50% [14,47]. In red wine technology, this parameter facilitates achieving the desired malic acid microbial stabilization before bottling [19]. However, in white wine production, *L. thermotolerans* strains may be selected to increase the malic acid content by approximately 7% [6], as most white wines do not require malic acid stabilization and could be of additional interest in highly productive, low acidic white grape varieties such as Airen. The production of glutathione up to the maximum concentration reported in wine for a strain of *L. thermotolerans* of 24 mg/L [23] can be established as the selection level. This may protect wine against oxidation and reduce the addition of sulfur dioxide. This can be of great interest in white wine production to protect the color or thiolic character. The highest reported concentration of polysaccharides in a sequential fermentation involving *L. thermotolerans* and *S. cerevisiae* is 700 mg/L [49].

Recent studies have shown that sequential fermentations involving *L. thermotolerans* reduce the final concentration of sulfur dioxide. Nowadays, there are consumers that demand wines with low concentrations of sulfur dioxide, such as people who suffer from asthma [50]. Additionally, European legislation is progressively reducing the allowed final concentration [50]. *L. thermotolerans* management could be an interesting strategy to reduce the final concentration of sulfur dioxide in wine. Additionally, this effect could synergize with the previously mentioned increases in glutathione [23]. The highest reported reduction compared to an *S. cerevisiae* control is 95% [14].

*L. thermotolerans* can be selected to show enzymatic activities that are strain-dependent, such as Lipase, CystineA, or β-Glucosidase [51]. Depending on the type of wine, these enzymatic activities could be of great interest. β-glucosidase could improve the final terpene concentration in terpenic varieties, such as Muscat of Alexandria, which has previously been studied in *L. thermotolerans* applications [70]. Previous studies have described, as an undesirable effect of *L. thermotolerans*, the production of high concentrations of fatty acids such as isovaleric acid, although the selection of *L. thermotolerans* strains without lipase activity may reduce that problem. Other non-*Saccharomyces* strains such as *Metschnikowia* or *Torulaspora* have previously been selected because of cysteine enzymatic activity in order to increase the content of thiols with high success [77]. The strains of *L. thermotolerans* have shown that cysteine enzymatic activity could also improve the content of thiols in thiolic varieties such as Sauvignon Blanc [15] or Riesling [53], which were previously studied in *L. thermotolerans* applications.

The selection of *L. thermotolerans* strains with pectinase, cellulose, xylanase, and glucanase enzymatic activity is of great interest in the production of wines with higher concentrations of polysaccharides. This could additionally enhance the color or improve the finning processes. The strain variability reported for these enzymatic activities could explain the contradictory results regarding the effect of *L. thermotolerans* species on wine and polysaccharide concentration [2].

Some studies have reported some *L. thermotolerans* strains to possess inhibitory effects over lactic bacteria [15]. These results show that compatibility with other microorganisms used in winemaking must be tested for every selected *L. thermotolerans* strain in order to avoid future problems on the industrial scale.

**Figure 2:**
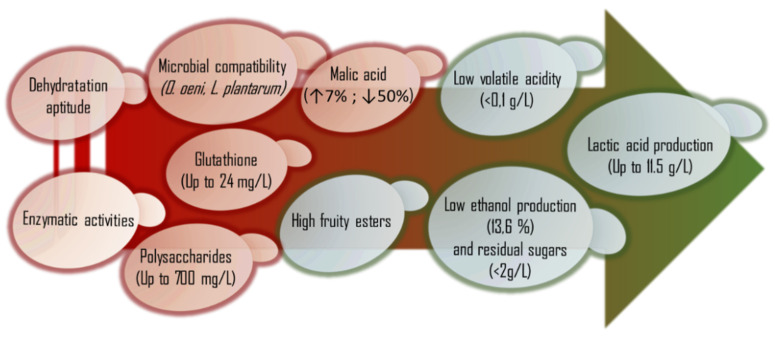
Summary of the proposed *Lachancea thermotolerans* selection parameters.

Although *L. thermotolerans* species possess many virtues, they have a problem when required to produce industrial dehydrated commercial products, as it is very sensitive to this process [78]. A new technology allows evaluating the biophysical stress responses of the *L. thermotolerans* strains during the dehydration process using synchrotron-FTIR microspectroscopy technology. This factor of dehydration aptitude has to be considered before commercializing a selected strain, as it can influence the cell viability of the final commercial product. Figure 2 summarizes the proposed selected parameters for *L. thermotolerans*.

## 9. Other *Lachancea* Species

### 9.1. Lachancea fermentati

*L. fermentati* shows higher H_2_S production than *L. thermotolerans*, varying from 25% to 50% higher [70]. *L. fermentati* showed higher SO_2_ tolerance and could proliferate at 20 mg/L of total SO_2_. *L. fermentati* could ferment up to 10.5% (*v*/*v*) in a single fermentation, while the *L. thermotolernas* controls fermented up to 10.1% (*v*/*v*). In sequential fermentations between *L. fermentati* or *L. thermotolerans* and *S. cerevisiae*, *L. fermentati* produced lower final concentrations in citric acid from 40% to 50% and higher final concentrations in acetic acid from 30% to 60%. Sequential fermentation of *L. fermetati* showed a lower final concentration of total esters of 95%. *L. fermetati* fermentations did not produce ethyl acetate, while the concentrations of other esters were very low. On the other side, sequential fermentations of *L. fermentati* showed higher final concentrations of total higher alcohols from 18% to 24%; the major differences took place for isobutanol and isoamyl alcohol. Additionally, sequential fermentations of *L. fermentati* also showed higher final concentrations in total volatile acids from 40% to 66%; the key differences took place for isobutyric acid.

### 9.2. Lachancea lanzarotensis

Although most studies use *L. thermotolerans* strains in fermentative studies, one study has tested *L. lanzarotensis* strains compared to others of *L. thermotolerans* and *L. fermentati* in wine fermentation of the Muscat of Alexandria variety [70]. The study only used two strains of *L. lanzarotensis* in must fermentation, but it is the only available scientific article to date. *L. lanzarotensis* possesses β-glucosidase enzymatic activity such as *L. thermotolerans*, but not β-xylosidase [70]. *L. lanzarotensis* is arbutin-positive but 4-methylumbelliferyl-β-d-glucoside-negative, while *L. thermotolerans* is positive for both.

*L. lanzarotensis* produces higher H_2_S levels, from 50% to 75%, than *L. thermotolerans* [70]. This study reported lower ethanol tolerance for the *L. lanzarotensis* strains that fermented wine up to 8.2% and 8.4% (*v*/*v*) in ethanol, while *L. thermotolerans* fermented up to 10.1% (*v*/*v*) in single fermentations. *L. lanzarotensis* fermentations in grape juice showed higher final concentrations in citric acid, succinic acid, acetic acid, and glycerol in 40–50%, 22–33%, 22–64%, and 20–18%, respectively.

In sequential fermentations between *L. lanzarotensis* or *L. thermotolerans* and *S. cerevisiae*, those of *L. lanzarotensis* showed a higher final concentration of total esters that varied from 10% to 22%, and the major differences took place for ethyl acetate. On the other side, sequential fermentations of *L. lanzarotensis* showed lower final concentrations of total higher alcohols from 9% to 14%, and the key differences took place for 1-propanol and isoamyl alcohol. Additionally, sequential fermentations of *L. lanzarotensis* also showed lower final concentrations in total volatile acids from 15% to 26%, and the fundamental differences took place for propionic and isobutyric acids. The final total terpene concentrations did not show significant differences, although *L. lanzarotensis* fermentations showed higher contents in citronellol by 22% to 27%.

## 10. Other *L. thermotolerans* Applications

### 10.1. Biocontrol and Inhibitory Effects

Former studies used *L. thermotolerans* as biological agents to protect grapes against fungal attacks [2], although the defense mechanism is not clearly known. A recent study [79] discovered four components of the class of heterocyclic alkaloids, three natural (4-Hydroxyquinoline, Xanthina, and Calistegina A3) and one synthetic (Clausehainanina C) from the crude extract of *L. thermotolerans*. These compounds are antimicrobial, antiproliferative, and inhibitory compounds. They have been tested as biocontrol agents in antagonistic tests of biological tests against phytopathogens that cause fungal and bacterial diseases in agricultural cultures. These results help understand the biocontrol activity of *L. thermotolerans* and the observed inhibitory effects observed in some fermentation studies.

### 10.2. Beer

Previous studies have suggested the application of *L. thermotolerans* in the beer industry [2] because its high fermentative power is high enough to complete the industrial process in a beverage with a lower ethanol content than wine. The last trends in *L. thermotolerans* application in beer production propose to select strains with low lactic acid-producing ability in order not to reduce the final pH of beer [80]. This results in a beer with lower ethanol, maltose, ester, and volatile acid contents, while the glycerol concentration increases.

## 11. Conclusions

*Lachancea thermotolerans* has already been established as the best bio-acidifier in the wine industry. This ability is of great interest for fighting climate change in modern winemaking. Recent studies have optimized and improved this ability while finding and developing other abilities that can improve the quality of wine. Several recent works have shown that it is possible to select better *L. thermotolerans* strains than those offered on the market. In addition to the classic selection of established parameters for *L. thermotolerans* such as lactic acid production, low acetic acid production, and high fermentative power, this review added new ones such as ethanol reduction, glutathione production, sulfur dioxide reduction, malic acid reduction or production, high polysaccharide production, compatibility with other species, color improvement, aroma enhancement, and enzymatic activities of enological interest. Those promising results show that in the future, there could be specific strains of *L.* *thermotolerans* able to ferment by themselves regular wines of moderate ethanol content and a great number of commercial strains adapted to different types of wine and winemaking objectives.

New combinations of *L. thermotolerans* with other yeasts different from *Saccharomyces cerevisiae*, such as *Schizosaccharomyces pombe*, and lactic bacteria such as *Oenococus oeni* or *Lactiplantibacillus plantarum* allow to produce stable wines from a microbiological point of view in less time just after alcoholic fermentation and to improve some of the quality parameters such as acetic acid, color, or diacetyl content. Combinations with other non-*Saccharomyces* strains such as *Torulaspora delbruecki* allow adding additional values to acidification, such as aroma enhancement.

Although the number of studies is limited, species of the *Lachancea* genus different from *L. thermotolerans* such as *L. lanzarotensis* or *L. fermentati* show interesting potential in fermentative industries.

Further studies must be performed regarding fermentation parameters, such as aeration or nitrogen nutrition. Studies that have compared some commercial *L. thermotolerans* strains between them have shown contradictory results, which show that there are important factors other than the strain parameter.

Despite the studies that have been carried out so far, the ecological role that *L. thermotolerans* plays in the enological ecosystem is still partially unknown. The molecular mechanisms in which the production of lactic acid is involved require further investigation. The regulatory routes and environmental conditions may define the increased activity of certain strains where lactic acid is concerned.

## Figures and Tables

**Table 1:** Summary of the variability reported for *L. thermotolerans* for several wine fermentation quality parameters.

Parameter	Benito et al., 2018 [2]	Hranilovic et al., 2018 [6]	Binati et al., 2019 [41]	Binati et al., 2020 [42]	Vaquero et al., 2020 [16]	Hranilovic et al., 2021 [14]	Zhang et al., 2021 [43]	Snyder et al., 2021 [15]
Fermentation modality	Pure	Pure	Pure	Sequential	Pure	Sequential	Sequential	Sequential
Ethanol (% *v*/*v*)	3.98–10.35	7.3–10.6	4.24–6.1	11.76–11.97	4.75–8		13.36–13.53	12.16–12.55
Glycerol (g/L)		3.9–8	3.97–4.99	4.67–5.30		9.6–11.6	6.95–8.1	6.4–7.2
Acetic acid (g/L)	0.1–0.58	0.06–0.32	0.03–0.23	0.19–0.26		0.29–0.54	0.24–0.37	<0.1–0.13
Polysaccharides (mg/L)	163–260							
Malic acid degradation (%)	10–25	(−7)–25				27–57		
Lactic acid production (g/L)	0.9–4.2	1.8–12	0.69–6.67	0.53–4.42	0.2–3	1–8.1	1.18–1.49	0.2–6.1
Pyruvic acid (mg/L)	20–28.5	13–78				60–170		
Succinic acid (mg/L)	287–438		403–597			2700–3900		
Acetaldehyde (mg/L)						11.7–18.7		
1-propanol (mg/L)	20.5–55.4					29,002–35,699		
Isobutanol (mg/L)	20.6–31.1					29,207–45,601		
Methionol (mg/L)	0.67–2.6							
Ethyl acetate (mg/L)	31–51					40,224–79,191	81,197–12,4867	
Ethyl lactate (mg/L)	5.5–23.2					10,617–185,507		
Acetoin (mg/L)	4.6–108							
Isovaleric acid (mg/L)	0.9–2							
Higher alcohols					180–275	430,882–595,281	342,891–400,396	

**Table 2:** Summary of studies that have compared different commercial *L. thermotolerans* strains in wine fermentation.

		*S. cerevisiae*	Levulia^TM^ AEB	Concerto^TM^ Hansen	Laktia^TM^ Lallemand
Ethanol % (*v*/*v*)	Hranilovic et al., 2021 [14]	16.5	16.2	15.6	15.7
	Snyder et al., 2021 [15]	12.71	12.38	12.55	12.24
	Vaquero et al., 2020 [16]	10.2	n.d.a.	8	6.2
Lactic acid (g/L)	Hranilovic et al., 2021 [14]	0.4	1	3.4	5.8
	Snyder et al., 2021 [15]	0.1	2.8	0.5	1.5
	Vaquero et al., 2020 [16]	0.15	n.d.a.	0.15–0.25	0.8–1.8
pH	Hranilovic et al., 2021 [14]	3.86	3.90	3.58	3.51
	Snyder et al., 2021 [15]	3.77	3.54	3.77	3.66
	Vaquero et al., 2020 [16]	3.7	n.d.a.	3.7	3.6
Total acidity (g/L)	Hranilovic et al., 2021 [14]	5	5.1	8.1	9.1
	Snyder et al., 2021 [15]	7.1	10.4	7.1	8.7
	Vaquero et al., 2020 [16]	3	n.d.a.	3	3.7
Acetic acid (g/L)	Hranilovic et al., 2021 [14]	0.15	0.47	0.49	0.45
	Snyder et al., 2021 [15]	0.17	<0.1	0.13	0.11
Malic acid (g/L)	Hranilovic et al., 2021 [14]	2.4	1.1	1.3	1.5
	Snyder et al., 2021 [15]	4.72	4.23	4.59	4.24
Total SO_2_ (mg/L)	Hranilovic et al., 2021 [14]	13.3	0.5	5.9	4.8
Higher Alcohols (mg/L)	Hranilovic et al., 2021 [14]	498	456	595	430
Ethyl esters (mg/L)	Hranilovic et al., 2021 [14]	40	57	138	163
Acetate esters (mg/L)	Hranilovic et al., 2021 [14]	1.73	1.80	2.65	2.11

n.d.a., no data available.

**Table 3:** Summary of sequential combinations between *L. thermotolerans* and *Saccharomyces* and their influence on the final lactic acid concentration, pH, and total acidity of the wine. The table reports the increases in lactic acid and total acidity and the reductions in pH that took place during alcoholic fermentation compared to the initial value of the grape juice before alcoholic fermentation.

Study	Lactic Acid (g/L) Increase	Total Acidity (g/L) Increase	pH Decrease
Kapsopoulou et al., 2007 [56]	1.8–5.13	2.04–5.2	0.13–0.3
Comitini et al., 2011 [57]	n.d.a.	1.8	0.23
Gobbi et al., 2013 [58]	6.38	5.1	0.26
Benito et al., 2015 [59]	1.22	n.d.a.	0.12
Benito et al., 2015 [60]	2.75	n.d.a.	0.22
Benito et al., 2016 [61]	3.18	n.d.a.	0.18
Balikci et al., 2016 [62]	n.d.a.	1.76–2	0.12
Benito et al., 2016 [17]	2.96	n.d.a.	0.21
Benito et al., 2017 [63]	2.44	n.d.a.	0.15
Chen et al., 2018 [64]	1.7	1.2	0.11
Dutraive et al., 2019 [53]	1.51	n.d.a.	0.2
Benito et al., 2019 [18]	1.63	n.d.a.	0.17
Morata et al., 2019 [65]	6.6	3.5	0.27
Blanco et al., 2020 [47]	7.1	5.7	0.3
Romani et al., 2020 [49]	0.78	1.58	0.26
Sgouros et al., 2020 [36]	10.4	12.08	0.29
Hranilovic et al., 2021 [14]	1–8.1	0.1–6.1	0.02–0.54
Snyder et al., 2021 [15]	0.2–6.1	0–6.2	0–0.33

n.d.a., no data available.

**Table 4:** Summary of the main *L. thermotolerans* combinations in winemaking during alcoholic fermentation, explaining their enological impact, advantages, and disadvantages.

Combined Species	Enological Impact	Advantages	Disadvantages
*Saccharomyces cerevisiae* Benito et al., 2018 [2]	Increase in acidification	pH reductions down to 0.5; ethanol reduction down to 1.6% (*v*/*v*)	Red wines need to perform malolactic fermentation after alcoholic fermentation
*Schizosaccharomyces pombe* Benito et al., 2020 [19]	Increase in acidification by *L. thermotolerans*; malic acid stabilization during alcoholic fermentation by *S. pombe*	Reduction in production hours; color increase; ethanol reduction down to 3% (*v*/*v*); biogenic amines control; ethyl carbamate control	*S. pombe* strain must be selected to produce low levels of acetic acid and sulfhidric acid
*Oenococus oeni* Snyder et al., 2021; Benito et al., 2021 [15,20]	Increase in acidification by *L. thermotolerans*; malic acid stabilization during alcoholic fermentation by *O. oeni*	Reduction in production hours; increase in color	Risk of an increase in acetic acid; inhibition of yeast development; biogenic amines production; ethyl carbamate production
*Lactiplantibacillus plantarum* Benito et al., 2021 [20]	Increase in acidification by *L. thermotolerans*; malic acid stabilization during alcoholic fermentation by *L. plantarum*	Reduction in production hours; increase in color; biogenic amines control; ethyl carbamate control	Limited capacity in very acid wines; compatibility between strains of *L. plantarum* and *L. thermotoerans*
Other non-*Saccharomyces* Hranilovic et al., 2018; Escribano et al., 2021; Vaquero et al., 2021 [21,51,67]	Additional value different from acidification	Aroma complexity; increase in glycerol; ethanol reduction; synergy	Low fermentative power of most non-*Saccharomyces*.

**Table 5:** Summary of the budding time, ripening time, productivity, and enological potential of grape varieties where *Lachancea thermotolerans* has been applied.

Study	Grape Variety	Budding Time	Ripening Time	Productivity	Enological Potential
Ciani et al., 2006; Binati et al., 2021 [23,68]	Pinot Grigio	Medium–early	Early	Medium–high	Vinified in white provides a perfumed dry, alcoholic wine of medium acidity; when macerated, the body increases
Gobbi et al., 2013 [58]	Sangiovese	n.d.a.	n.d.a.	n.d.a.	n.d.a.
Benito et al., 2015; Dutraive et al., 2019 [53,59]	Riesling	Medium	Medium	Medium–low	Provides aromatic white wines with greenish reflections
Benito et al., 2015, 2016, 2017, 2018, 2019; Morata et al., 2019 [2,17,18,60,63,65]	Tempranillo	Medium–early	Medium–early	Medium	Fruity wines of intense and stable color, high alcoholic graduation, and medium–low acidity depending on the degree of ripening
Beckner Whitener et al., 2015, 2017 [72,73]	Syrah	Medium–late	Medium	Medium	Produces aromatic ruby red and purple wines, good structure, alcoholic, complex, and tannic
Benito et al., 2016; Vaquero et al., 2020 [16,61]	Airen	Late	Late	High	Neutral and alcoholic wine of medium–low quality due to its high productivity; the acidity level is low
Balikci et al., 2016 [62]	Emir	n.d.a.	n.d.a.	n.d.a.	n.d.a.
Beckner Whitener et al., 2016; Snyder 2021 [15,74]	Sauvignon Blanc	Medium–early	Medium	Medium–low	Provides aromatic wines with a thiolic character
Shekhawat et al., 2018 [22]	Chardonnay	Early	Early	Medium–high	Provides wines with varietal flavor, yellow with golden reflections, and alcoholic
Porter et al., 2019 [70]	Muscat of Alexandria	Medium–late	Medium–late	Medium	Provides yellow wines with a golden character and a strong terpenic aroma
Castrillo et al., 2019 [71]	Treixadura	Medium–late	Medium	High	Produces wines of good quality and moderate alcohol and acidity content in Atlantic regions
Blanco et al., 2020 [47]	Mencia	Medium–late	Early	Medium–high	Purple wines with elegant aromas and a good balance of alcohol and acidity
Hranilovic et al., 2021 [14]	Merlot	Medium	Medium	Medium–high	Provides fine fruity wines with an intense ruby red color, alcoholic, and with a tendency of low acidity
Escribano-Viana et al., 2021 [51]	Viura	Late	Late	High	Neutral and alcoholic wine of medium–low quality due to its high productivity
Korenika et al., 2021 [44]	Trnjak, Babic, Blatina, and Frankovka	n.d.a.	n.d.a.	n.d.a.	n.d.a.
González-Alonso et al., 2021 [75]					

Source: Own elaboration adapted and summarized from the book “Vine Varieties: Register of Commercial Varieties” published by the Spanish Ministry of Agriculture, Fisheries and Food, according to the study of several grape varieties and clones planted in the Polytechnic University of Madrid experimental crops under same conditions [76]. n.d.a., no data available. The varieties without data were not planted in the grape variety collection plantation of the Polytechnic University of Madrid.

## Data Availability

Not applicable.
